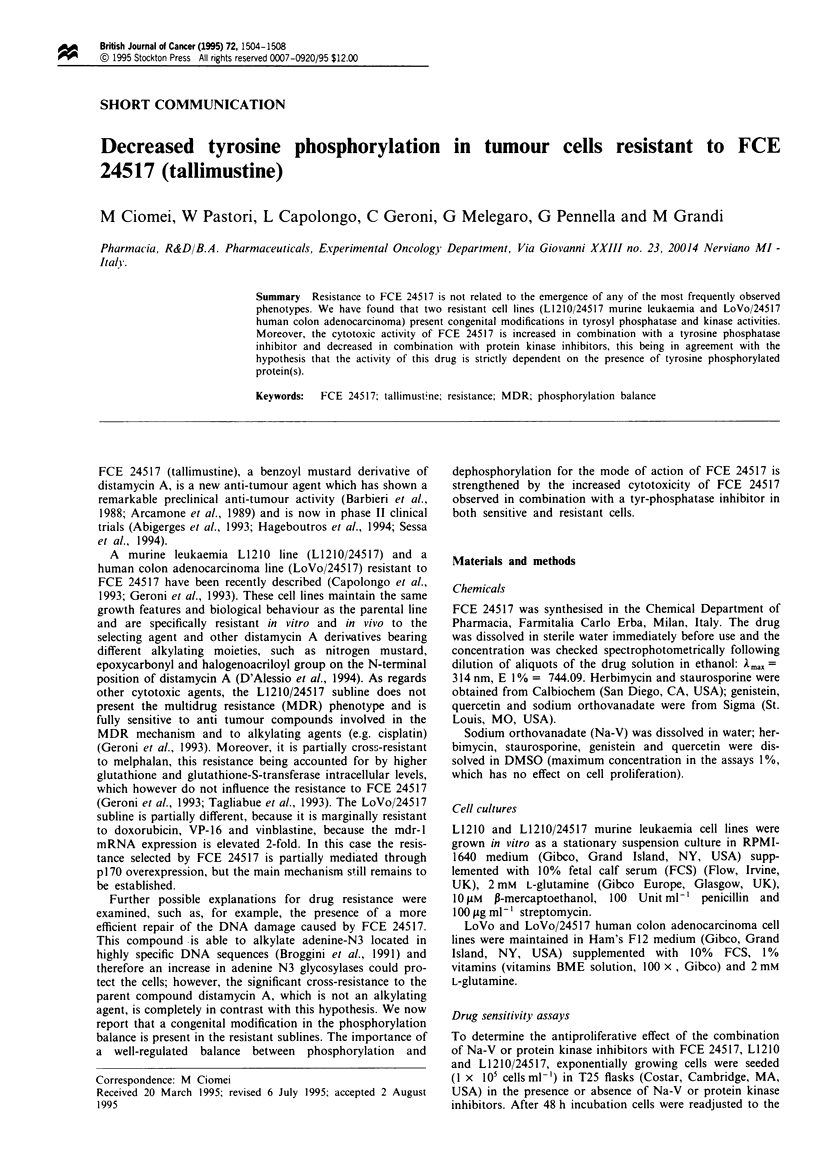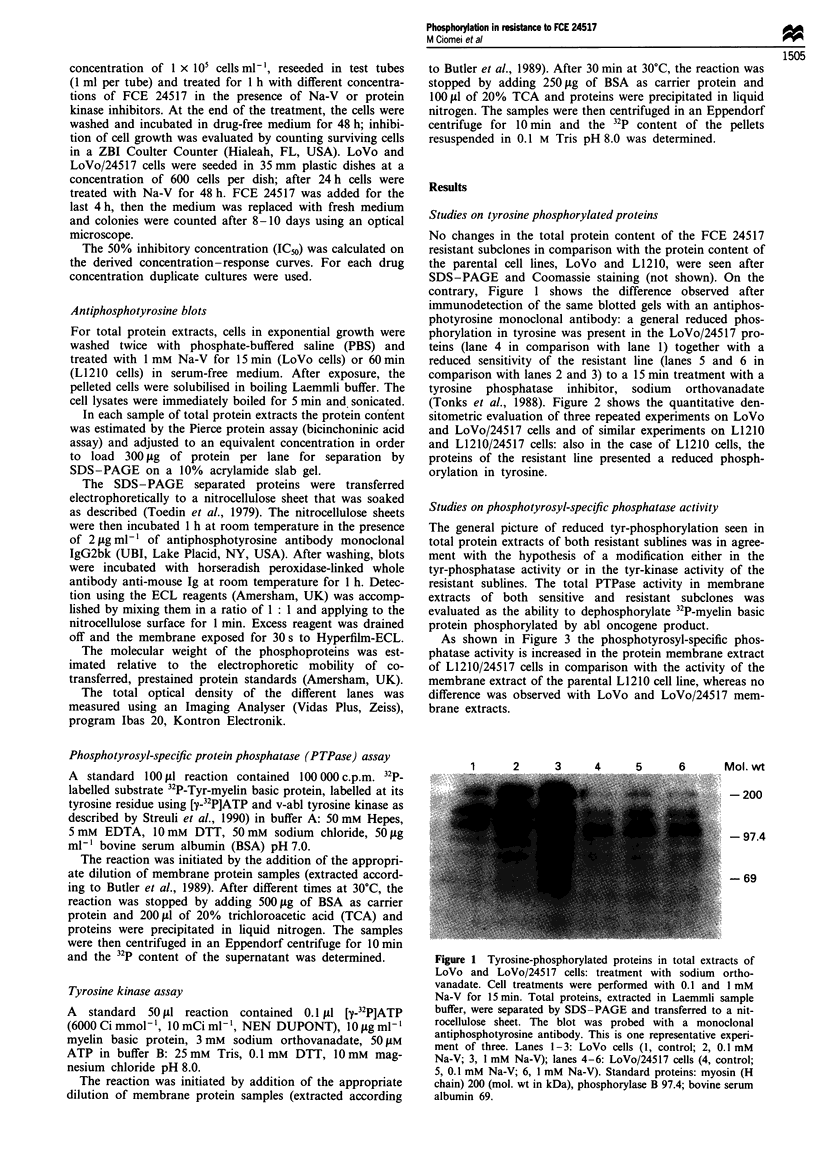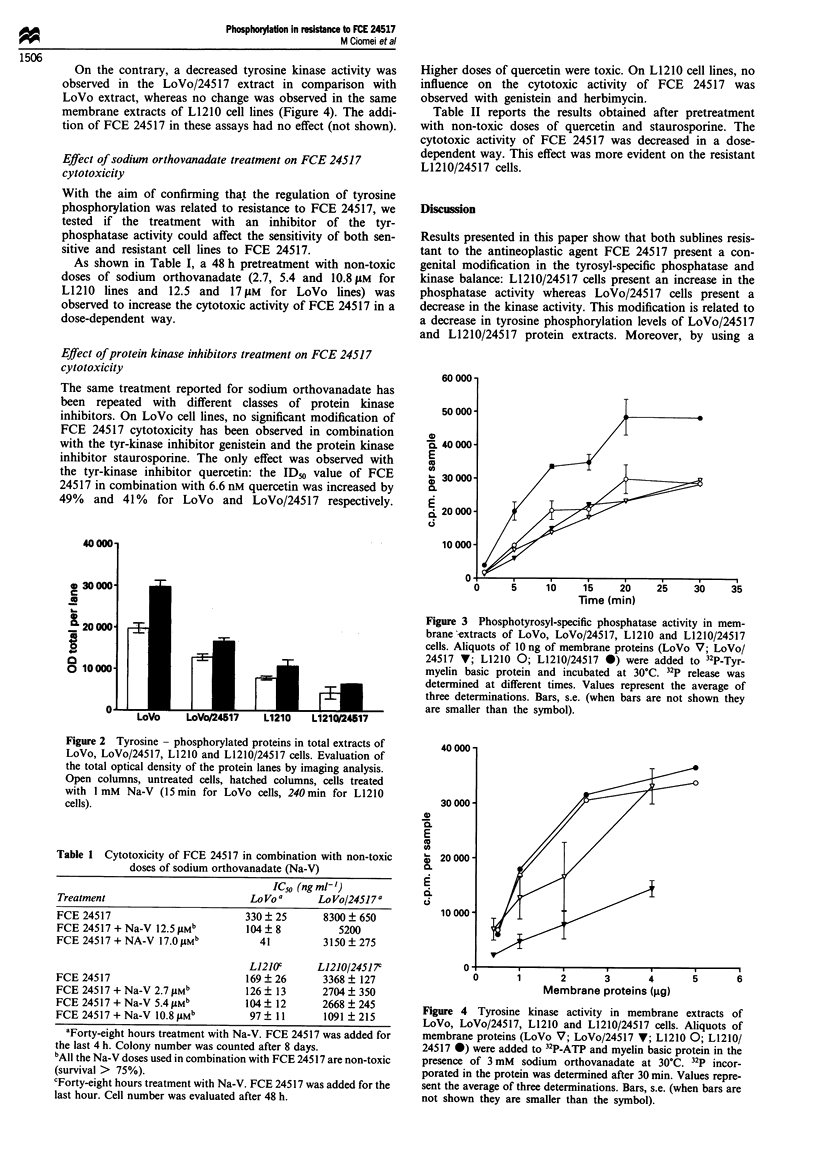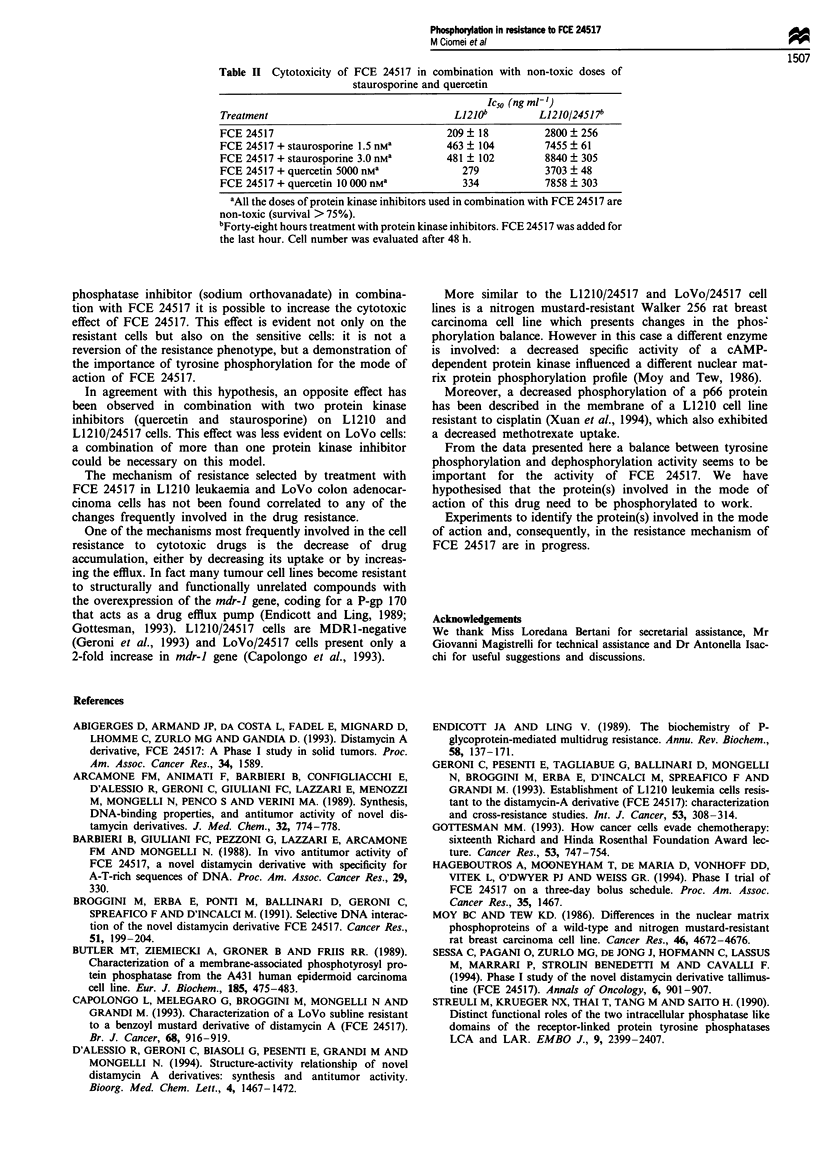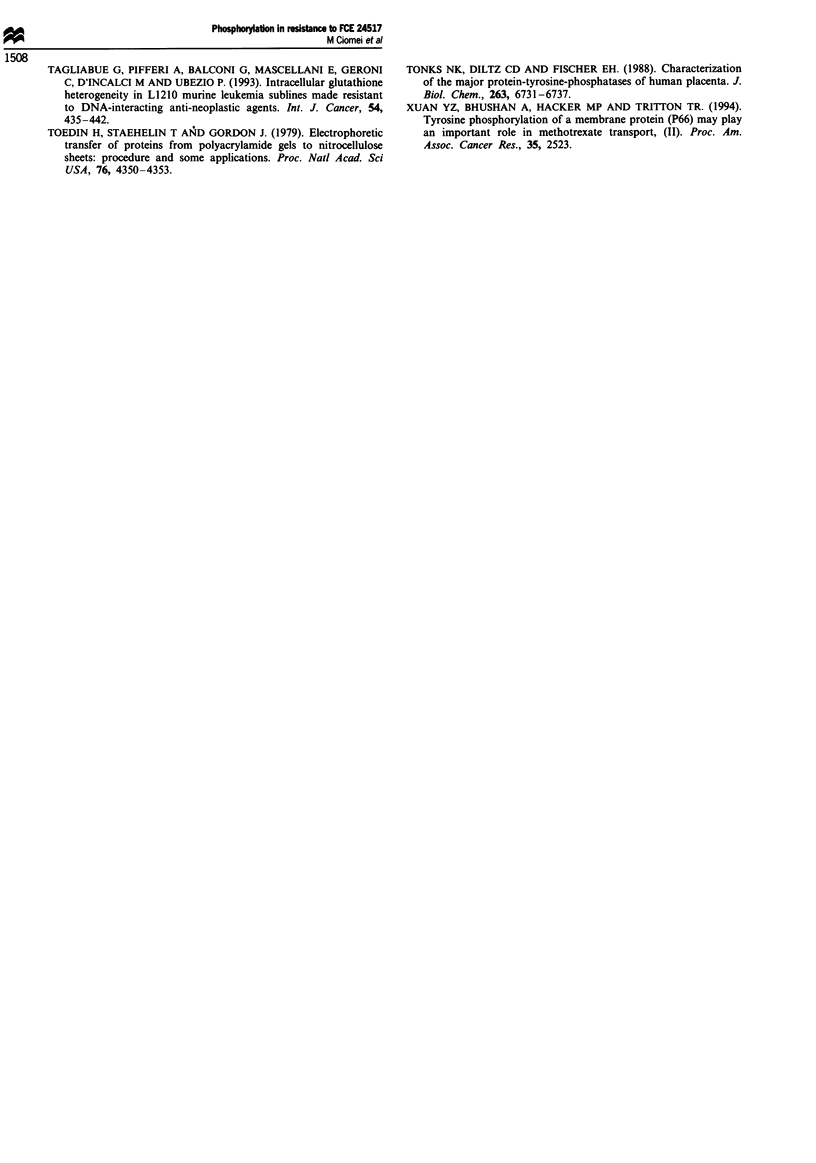# Decreased tyrosine phosphorylation in tumour cells resistant to FCE 24517 (tallimustine).

**DOI:** 10.1038/bjc.1995.537

**Published:** 1995-12

**Authors:** M. Ciomei, W. Pastori, L. Capolongo, C. Geroni, G. Melegaro, G. Pennella, M. Grandi

**Affiliations:** Pharmacia, R&D/B.A. Pharmaceuticals, Experimental Oncology Department, Nerviano MI-Italy.

## Abstract

**Images:**


British Journal of Cancer (1995) 72, 1504-1508
© 1995 Stockton Press All rights reserved 0007-0920/95 $12.00

SHORT COMMUNICATION

### Decreased tyrosine phosphorylation in tumour cells resistant to FCE 24517 (tallimustine)

M Ciomei, W Pastori, L Capolongo, C Geroni, G Melegaro, G Pennella and M Grandi

*Pharmacia, R&D/B.A. Pharmaceuticals, Experimental Oncology Department, Via Giovanni XXIII no. 23, 20014 Nerviano MI - Italy.*

**Summary** Resistance to FCE 24517 is not related to the emergence of any of the most frequently observed phenotypes. We have found that two resistant cell lines (L1210/24517 murine leukaemia and LoVo/24517 human colon adenocarcinoma) present congenital modifications in tyrosyl phosphatase and kinase activities. Moreover, the cytotoxic activity of FCE 24517 is increased in combination with a tyrosine phosphatase inhibitor and decreased in combination with protein kinase inhibitors, this being in agreement with the hypothesis that the activity of this drug is strictly dependent on the presence of tyrosine phosphorylated protein(s).

**Keywords:** FCE 24517; tallimustine; resistance; MDR; phosphorylation balance

FCE 24517 (tallimustine), a benzoyl mustard derivative of distamycin A, is a new anti-tumour agent which has shown a remarkable preclinical anti-tumour activity (Barbieri *et al.*, 1988; Arcamone *et al.*, 1989) and is now in phase II clinical trials (Abigerges *et al.*, 1993; Hageboutros *et al.*, 1994; Sessa *et al.*, 1994).

A murine leukaemia L1210 line (L1210/24517) and a human colon adenocarcinoma line (LoVo/24517) resistant to FCE 24517 have been recently described (Capolongo *et al.*, 1993; Geroni *et al.*, 1993). These cell lines maintain the same growth features and biological behaviour as the parental line and are specifically resistant *in vitro* and *in vivo* to the selecting agent and other distamycin A derivatives bearing different alkylating moieties, such as nitrogen mustard, epoxycarbonyl and halogenoacriloyl group on the N-terminal position of distamycin A (D'Alessio *et al.*, 1994). As regards other cytotoxic agents, the L1210/24517 subline does not present the multidrug resistance (MDR) phenotype and is fully sensitive to anti tumour compounds involved in the MDR mechanism and to alkylating agents (e.g. cisplatin) (Geroni *et al.*, 1993). Moreover, it is partially cross-resistant to melphalan, this resistance being accounted for by higher glutathione and glutathione-S-transferase intracellular levels, which however do not influence the resistance to FCE 24517 (Geroni *et al.*, 1993; Tagliabue *et al.*, 1993). The LoVo/24517 subline is partially different, because it is marginally resistant to doxorubicin, VP-16 and vinblastine, because the mdr-1 mRNA expression is elevated 2-fold. In this case the resistance selected by FCE 24517 is partially mediated through p170 overexpression, but the main mechanism still remains to be established.

Further possible explanations for drug resistance were examined, such as, for example, the presence of a more efficient repair of the DNA damage caused by FCE 24517. This compound is able to alkylate adenine-N3 located in highly specific DNA sequences (Broggini *et al.*, 1991) and therefore an increase in adenine N3 glycosylases could protect the cells; however, the significant cross-resistance to the parent compound distamycin A, which is not an alkylating agent, is completely in contrast with this hypothesis. We now report that a congenital modification in the phosphorylation balance is present in the resistant sublines. The importance of a well-regulated balance between phosphorylation and dephosphorylation for the mode of action of FCE 24517 is strengthened by the increased cytotoxicity of FCE 24517 observed in combination with a tyr-phosphatase inhibitor in both sensitive and resistant cells.

Correspondence: M Ciomei
Received 20 March 1995; revised 6 July 1995; accepted 2 August 1995

### Materials and methods

### *Chemicals*

FCE 24517 was synthesised in the Chemical Department of Pharmacia, Farmitalia Carlo Erba, Milan, Italy. The drug was dissolved in sterile water immediately before use and the concentration was checked spectrophotometrically following dilution of aliquots of the drug solution in ethanol: $\lambda_{max} =$ 314 nm, E 1% = 744.09. Herbimycin and staurosporine were obtained from Calbiochem (San Diego, CA, USA); genistein, quercetin and sodium orthovanadate were from Sigma (St. Louis, MO, USA).

Sodium orthovanadate (Na-V) was dissolved in water; herbimycin, staurosporine, genistein and quercetin were dissolved in DMSO (maximum concentration in the assays 1%, which has no effect on cell proliferation).

### *Cell cultures*

L1210 and L1210/24517 murine leukaemia cell lines were grown *in vitro* as a stationary suspension culture in RPMI-1640 medium (Gibco, Grand Island, NY, USA) supplemented with 10% fetal calf serum (FCS) (Flow, Irvine, UK), 2 mM L-glutamine (Gibco Europe, Glasgow, UK), 10 $\mu$M $\beta$-mercaptoethanol, 100 Unit $ml^{-1}$ penicillin and 100 $\mu g\ ml^{-1}$ streptomycin.

LoVo and LoVo/24517 human colon adenocarcinoma cell lines were maintained in Ham's F12 medium (Gibco, Grand Island, NY, USA) supplemented with 10% FCS, 1% vitamins (vitamins BME solution, 100 ×, Gibco) and 2 mM L-glutamine.

### *Drug sensitivity assays*

To determine the antiproliferative effect of the combination of Na-V or protein kinase inhibitors with FCE 24517, L1210 and L1210/24517, exponentially growing cells were seeded ($1 \times 10^5$ cells $ml^{-1}$) in T25 flasks (Costar, Cambridge, MA, USA) in the presence or absence of Na-V or protein kinase inhibitors. After 48 h incubation cells were readjusted to the concentration of $1 \times 10^5$ cells $ml^{-1}$, reseeded in test tubes (1 ml per tube) and treated for 1 h with different concentrations of FCE 24517 in the presence of Na-V or protein kinase inhibitors. At the end of the treatment, the cells were washed and incubated in drug-free medium for 48 h; inhibition of cell growth was evaluated by counting surviving cells in a ZBI Coulter Counter (Hialeah, FL, USA). LoVo and LoVo/24517 cells were seeded in 35 mm plastic dishes at a concentration of 600 cells per dish; after 24 h cells were treated with Na-V for 48 h. FCE 24517 was added for the last 4 h, then the medium was replaced with fresh medium and colonies were counted after 8–10 days using an optical microscope.

The 50% inhibitory concentration ($IC_{50}$) was calculated on the derived concentration–response curves. For each drug concentration duplicate cultures were used.

### *Antiphosphotyrosine blots*

For total protein extracts, cells in exponential growth were washed twice with phosphate-buffered saline (PBS) and treated with 1 mM Na-V for 15 min (LoVo cells) or 60 min (L1210 cells) in serum-free medium. After exposure, the pelleted cells were solubilised in boiling Laemmli buffer. The cell lysates were immediately boiled for 5 min and sonicated.

In each sample of total protein extracts the protein content was estimated by the Pierce protein assay (bicinchoninic acid assay) and adjusted to an equivalent concentration in order to load 300 μg of protein per lane for separation by SDS–PAGE on a 10% acrylamide slab gel.

The SDS–PAGE separated proteins were transferred electrophoretically to a nitrocellulose sheet that was soaked as described (Toedin *et al.*, 1979). The nitrocellulose sheets were then incubated 1 h at room temperature in the presence of 2 μg $ml^{-1}$ of antiphosphotyrosine antibody monoclonal IgG2bk (UBI, Lake Placid, NY, USA). After washing, blots were incubated with horseradish peroxidase-linked whole antibody anti-mouse Ig at room temperature for 1 h. Detection using the ECL reagents (Amersham, UK) was accomplished by mixing them in a ratio of 1 : 1 and applying to the nitrocellulose surface for 1 min. Excess reagent was drained off and the membrane exposed for 30 s to Hyperfilm-ECL.

The molecular weight of the phosphoproteins was estimated relative to the electrophoretic mobility of co-transferred, prestained protein standards (Amersham, UK).

The total optical density of the different lanes was measured using an Imaging Analyser (Vidas Plus, Zeiss), program Ibas 20, Kontron Electronik.

### *Phosphotyrosyl-specific protein phosphatase (PTPase) assay*

A standard 100 μl reaction contained 100 000 c.p.m. $^{32}P$-labelled substrate $^{32}P$-Tyr-myelin basic protein, labelled at its tyrosine residue using [$\gamma$-$^{32}P$]ATP and v-abl tyrosine kinase as described by Streuli *et al.*, 1990) in buffer A: 50 mM Hepes, 5 mM EDTA, 10 mM DTT, 50 mM sodium chloride, 50 μg $ml^{-1}$ bovine serum albumin (BSA) pH 7.0.

The reaction was initiated by the addition of the appropriate dilution of membrane protein samples (extracted according to Butler *et al.*, 1989). After different times at 30°C, the reaction was stopped by adding 500 μg of BSA as carrier protein and 200 μl of 20% trichloroacetic acid (TCA) and proteins were precipitated in liquid nitrogen. The samples were then centrifuged in an Eppendorf centrifuge for 10 min and the $^{32}P$ content of the supernatant was determined.

### *Tyrosine kinase assay*

A standard 50 μl reaction contained 0.1 μl [$\gamma$-$^{32}P$]ATP (6000 Ci $mmol^{-1}$, 10 mCi $ml^{-1}$, NEN DUPONT), 10 μg $ml^{-1}$ myelin basic protein, 3 mM sodium orthovanadate, 50 μM ATP in buffer B: 25 mM Tris, 0.1 mM DTT, 10 mM magnesium chloride pH 8.0.

The reaction was initiated by addition of the appropriate dilution of membrane protein samples (extracted according to Butler *et al.*, 1989). After 30 min at 30°C, the reaction was stopped by adding 250 μg of BSA as carrier protein and 100 μl of 20% TCA and proteins were precipitated in liquid nitrogen. The samples were then centrifuged in an Eppendorf centrifuge for 10 min and the $^{32}P$ content of the pellets resuspended in 0.1 M Tris pH 8.0 was determined.

### Results

### *Studies on tyrosine phosphorylated proteins*

No changes in the total protein content of the FCE 24517 resistant subclones in comparison with the protein content of the parental cell lines, LoVo and L1210, were seen after SDS–PAGE and Coomassie staining (not shown). On the contrary, Figure 1 shows the difference observed after immunodetection of the same blotted gels with an antiphosphotyrosine monoclonal antibody: a general reduced phosphorylation in tyrosine was present in the LoVo/24517 proteins (lane 4 in comparison with lane 1) together with a reduced sensitivity of the resistant line (lanes 5 and 6 in comparison with lanes 2 and 3) to a 15 min treatment with a tyrosine phosphatase inhibitor, sodium orthovanadate (Tonks *et al.*, 1988). Figure 2 shows the quantitative densitometric evaluation of three repeated experiments on LoVo and LoVo/24517 cells and of similar experiments on L1210 and L1210/24517 cells: also in the case of L1210 cells, the proteins of the resistant line presented a reduced phosphorylation in tyrosine.

### *Studies on phosphotyrosyl-specific phosphatase activity*

The general picture of reduced tyr-phosphorylation seen in total protein extracts of both resistant sublines was in agreement with the hypothesis of a modification either in the tyr-phosphatase activity or in the tyr-kinase activity of the resistant sublines. The total PTPase activity in membrane extracts of both sensitive and resistant subclones was evaluated as the ability to dephosphorylate $^{32}P$-myelin basic protein phosphorylated by abl oncogene product.

As shown in Figure 3 the phosphotyrosyl-specific phosphatase activity is increased in the protein membrane extract of L1210/24517 cells in comparison with the activity of the membrane extract of the parental L1210 cell line, whereas no difference was observed with LoVo and LoVo/24517 membrane extracts.

**Figure 1** Tyrosine-phosphorylated proteins in total extracts of LoVo and LoVo/24517 cells: treatment with sodium orthovanadate. Cell treatments were performed with 0.1 and 1 mM Na-V for 15 min. Total proteins, extracted in Laemmli sample buffer, were separated by SDS–PAGE and transferred to a nitrocellulose sheet. The blot was probed with a monoclonal antiphosphotyrosine antibody. This is one representative experiment of three. Lanes 1–3: LoVo cells (1, control; 2, 0.1 mM Na-V; 3, 1 mM Na-V); lanes 4–6: LoVo/24517 cells (4, control; 5, 0.1 mM Na-V; 6, 1 mM Na-V). Standard proteins: myosin (H chain) 200 (mol. wt in kDa), phosphorylase B 97.4; bovine serum albumin 69.

On the contrary, a decreased tyrosine kinase activity was observed in the LoVo/24517 extract in comparison with LoVo extract, whereas no change was observed in the same membrane extracts of L1210 cell lines (Figure 4). The addition of FCE 24517 in these assays had no effect (not shown).

### *Effect of sodium orthovanadate treatment on FCE 24517 cytotoxicity*

With the aim of confirming that the regulation of tyrosine phosphorylation was related to resistance to FCE 24517, we tested if the treatment with an inhibitor of the tyr-phosphatase activity could affect the sensitivity of both sensitive and resistant cell lines to FCE 24517.

As shown in Table I, a 48 h pretreatment with non-toxic doses of sodium orthovanadate (2.7, 5.4 and 10.8 μM for L1210 lines and 12.5 and 17 μM for LoVo lines) was observed to increase the cytotoxic activity of FCE 24517 in a dose-dependent way.

### *Effect of protein kinase inhibitors treatment on FCE 24517 cytotoxicity*

The same treatment reported for sodium orthovanadate has been repeated with different classes of protein kinase inhibitors. On LoVo cell lines, no significant modification of FCE 24517 cytotoxicity has been observed in combination with the tyr-kinase inhibitor genistein and the protein kinase inhibitor staurosporine. The only effect was observed with the tyr-kinase inhibitor quercetin: the $ID_{50}$ value of FCE 24517 in combination with 6.6 nM quercetin was increased by 49% and 41% for LoVo and LoVo/24517 respectively.

**Figure 2** Tyrosine – phosphorylated proteins in total extracts of LoVo, LoVo/24517, L1210 and L1210/24517 cells. Evaluation of the total optical density of the protein lanes by imaging analysis. Open columns, untreated cells, hatched columns, cells treated with 1 mM Na-V (15 min for LoVo cells, *240* min for L1210 cells).

**Table 1** Cytotoxicity of FCE 24517 in combination with non-toxic doses of sodium orthovanadate (Na-V)

| *Treatment* | *$IC_{50}$ (ng $ml^{-1}$)* *LoVo*[a] | *LoVo/24517*[a] |
|---|---|---|
| FCE 24517 | 330 ± 25 | 8300 ± 650 |
| FCE 24517 + Na-V 12.5 μM[b] | 104 ± 8 | 5200 |
| FCE 24517 + NA-V 17.0 μM[b] | 41 | 3150 ± 275 |
| | *L1210*[c] | *L1210/24517*[c] |
| FCE 24517 | 169 ± 26 | 3368 ± 127 |
| FCE 24517 + Na-V 2.7 μM[b] | 126 ± 13 | 2704 ± 350 |
| FCE 24517 + Na-V 5.4 μM[b] | 104 ± 12 | 2668 ± 245 |
| FCE 24517 + Na-V 10.8 μM[b] | 97 ± 11 | 1091 ± 215 |

[a]Forty-eight hours treatment with Na-V. FCE 24517 was added for the last 4 h. Colony number was counted after 8 days.
[b]All the Na-V doses used in combination with FCE 24517 are non-toxic (survival > 75%).
[c]Forty-eight hours treatment with Na-V. FCE 24517 was added for the last hour. Cell number was evaluated after 48 h.

Higher doses of quercetin were toxic. On L1210 cell lines, no influence on the cytotoxic activity of FCE 24517 was observed with genistein and herbimycin.

Table II reports the results obtained after pretreatment with non-toxic doses of quercetin and staurosporine. The cytotoxic activity of FCE 24517 was decreased in a dose-dependent way. This effect was more evident on the resistant L1210/24517 cells.

### Discussion

Results presented in this paper show that both sublines resistant to the antineoplastic agent FCE 24517 present a congenital modification in the tyrosyl-specific phosphatase and kinase balance: L1210/24517 cells present an increase in the phosphatase activity whereas LoVo/24517 cells present a decrease in the kinase activity. This modification is related to a decrease in tyrosine phosphorylation levels of LoVo/24517 and L1210/24517 protein extracts. Moreover, by using a

**Figure 3** Phosphotyrosyl-specific phosphatase activity in membrane extracts of LoVo, LoVo/24517, L1210 and L1210/24517 cells. Aliquots of 10 ng of membrane proteins (LoVo ▽; LoVo/24517 ▼; L1210 ○; L1210/24517 ●) were added to $^{32}$P-Tyr-myelin basic protein and incubated at 30°C. $^{32}$P release was determined at different times. Values represent the average of three determinations. Bars, s.e. (when bars are not shown they are smaller than the symbol).

**Figure 4** Tyrosine kinase activity in membrane extracts of LoVo, LoVo/24517, L1210 and L1210/24517 cells. Aliquots of membrane proteins (LoVo ▽; LoVo/24517 ▼; L1210 ○; L1210/24517 ●) were added to $^{32}$P-ATP and myelin basic protein in the presence of 3 mM sodium orthovanadate at 30°C. $^{32}$P incorporated in the protein was determined after 30 min. Values represent the average of three determinations. Bars, s.e. (when bars are not shown they are smaller than the symbol).

**Table II** Cytotoxicity of FCE 24517 in combination with non-toxic doses of staurosporine and quercetin

| *Treatment* | *$IC_{50}$ (ng ml$^{-1}$)* L1210[b] | L1210/24517[b] |
|---|---|---|
| FCE 24517 | 209 ± 18 | 2800 ± 256 |
| FCE 24517 + staurosporine 1.5 nM[a] | 463 ± 104 | 7455 ± 61 |
| FCE 24517 + staurosporine 3.0 nM[a] | 481 ± 102 | 8840 ± 305 |
| FCE 24517 + quercetin 5000 nM[a] | 279 | 3703 ± 48 |
| FCE 24517 + quercetin 10 000 nM[a] | 334 | 7858 ± 303 |

[a]All the doses of protein kinase inhibitors used in combination with FCE 24517 are non-toxic (survival > 75%).
[b]Forty-eight hours treatment with protein kinase inhibitors. FCE 24517 was added for the last hour. Cell number was evaluated after 48 h.

phosphatase inhibitor (sodium orthovanadate) in combination with FCE 24517 it is possible to increase the cytotoxic effect of FCE 24517. This effect is evident not only on the resistant cells but also on the sensitive cells: it is not a reversion of the resistance phenotype, but a demonstration of the importance of tyrosine phosphorylation for the mode of action of FCE 24517.

In agreement with this hypothesis, an opposite effect has been observed in combination with two protein kinase inhibitors (quercetin and staurosporine) on L1210 and L1210/24517 cells. This effect was less evident on LoVo cells: a combination of more than one protein kinase inhibitor could be necessary on this model.

The mechanism of resistance selected by treatment with FCE 24517 in L1210 leukaemia and LoVo colon adenocarcinoma cells has not been found correlated to any of the changes frequently involved in the drug resistance.

One of the mechanisms most frequently involved in the cell resistance to cytotoxic drugs is the decrease of drug accumulation, either by decreasing its uptake or by increasing the efflux. In fact many tumour cell lines become resistant to structurally and functionally unrelated compounds with the overexpression of the *mdr-1* gene, coding for a P-gp 170 that acts as a drug efflux pump (Endicott and Ling, 1989; Gottesman, 1993). L1210/24517 cells are MDR1-negative (Geroni *et al.*, 1993) and LoVo/24517 cells present only a 2-fold increase in *mdr-1* gene (Capolongo *et al.*, 1993).

More similar to the L1210/24517 and LoVo/24517 cell lines is a nitrogen mustard-resistant Walker 256 rat breast carcinoma cell line which presents changes in the phosphorylation balance. However in this case a different enzyme is involved: a decreased specific activity of a cAMP-dependent protein kinase influenced a different nuclear matrix protein phosphorylation profile (Moy and Tew, 1986).

Moreover, a decreased phosphorylation of a p66 protein has been described in the membrane of a L1210 cell line resistant to cisplatin (Xuan *et al.*, 1994), which also exhibited a decreased methotrexate uptake.

From the data presented here a balance between tyrosine phosphorylation and dephosphorylation activity seems to be important for the activity of FCE 24517. We have hypothesised that the protein(s) involved in the mode of action of this drug need to be phosphorylated to work.

Experiments to identify the protein(s) involved in the mode of action and, consequently, in the resistance mechanism of FCE 24517 are in progress.

**Acknowledgements**
We thank Miss Loredana Bertani for secretarial assistance, Mr Giovanni Magistrelli for technical assistance and Dr Antonella Isacchi for useful suggestions and discussions.

**References**

ABIGERGES D, ARMAND JP, DA COSTA L, FADEL E, MIGNARD D, LHOMME C, ZURLO MG AND GANDIA D. (1993). Distamycin A derivative, FCE 24517: A Phase I study in solid tumors. *Proc. Am. Assoc. Cancer Res.*, **34**, 1589.

ARCAMONE FM, ANIMATI F, BARBIERI B, CONFIGLIACCHI E, D'ALESSIO R, GERONI C, GIULIANI FC, LAZZARI E, MENOZZI M, MONGELLI N, PENCO S AND VERINI MA. (1989). Synthesis, DNA-binding properties, and antitumor activity of novel distamycin derivatives. *J. Med. Chem.*, **32**, 774-778.

BARBIERI B, GIULIANI FC, PEZZONI G, LAZZARI E, ARCAMONE FM AND MONGELLI N. (1988). In vivo antitumor activity of FCE 24517, a novel distamycin derivative with specificity for A-T-rich sequences of DNA. *Proc. Am. Assoc. Cancer Res.*, **29**, 330.

BROGGINI M, ERBA E, PONTI M, BALLINARI D, GERONI C, SPREAFICO F AND D'INCALCI M. (1991). Selective DNA interaction of the novel distamycin derivative FCE 24517. *Cancer Res.*, **51**, 199-204.

BUTLER MT, ZIEMIECKI A, GRONER B AND FRIIS RR. (1989). Characterization of a membrane-associated phosphotyrosyl protein phosphatase from the A431 human epidermoid carcinoma cell line. *Eur. J. Biochem.*, **185**, 475-483.

CAPOLONGO L, MELEGARO G, BROGGINI M, MONGELLI N AND GRANDI M. (1993). Characterization of a LoVo subline resistant to a benzoyl mustard derivative of distamycin A (FCE 24517). *Br. J. Cancer*, **68**, 916-919.

D'ALESSIO R, GERONI C, BIASOLI G, PESENTI E, GRANDI M AND MONGELLI N. (1994). Structure-activity relationship of novel distamycin A derivatives: synthesis and antitumor activity. *Bioorg. Med. Chem. Lett.*, **4**, 1467-1472.

ENDICOTT JA AND LING V. (1989). The biochemistry of P-glycoprotein-mediated multidrug resistance. *Annu. Rev. Biochem.*, **58**, 137-171.

GERONI C, PESENTI E, TAGLIABUE G, BALLINARI D, MONGELLI N, BROGGINI M, ERBA E, D'INCALCI M, SPREAFICO F AND GRANDI M. (1993). Establishment of L1210 leukemia cells resistant to the distamycin-A derivative (FCE 24517): characterization and cross-resistance studies. *Int. J. Cancer*, **53**, 308-314.

GOTTESMAN MM. (1993). How cancer cells evade chemotherapy: sixteenth Richard and Hinda Rosenthal Foundation Award lecture. *Cancer Res.*, **53**, 747-754.

HAGEBOUTROS A, MOONEYHAM T, DE MARIA D, VONHOFF DD, VITEK L, O'DWYER PJ AND WEISS GR. (1994). Phase I trial of FCE 24517 on a three-day bolus schedule. *Proc. Am. Assoc. Cancer Res.*, **35**, 1467.

MOY BC AND TEW KD. (1986). Differences in the nuclear matrix phosphoproteins of a wild-type and nitrogen mustard-resistant rat breast carcinoma cell line. *Cancer Res.*, **46**, 4672-4676.

SESSA C, PAGANI O, ZURLO MG, DE JONG J, HOFMANN C, LASSUS M, MARRARI P, STROLIN BENEDETTI M AND CAVALLI F. (1994). Phase I study of the novel distamycin derivative tallimustine (FCE 24517). *Annals of Oncology*, **6**, 901-907.

STREULI M, KRUEGER NX, THAI T, TANG M AND SAITO H. (1990). Distinct functional roles of the two intracellular phosphatase like domains of the receptor-linked protein tyrosine phosphatases LCA and LAR. *EMBO J.*, **9**, 2399-2407.

TAGLIABUE G, PIFFERI A, BALCONI G, MASCELLANI E, GERONI C, D'INCALCI M AND UBEZIO P. (1993). Intracellular glutathione heterogeneity in L1210 murine leukemia sublines made resistant to DNA-interacting anti-neoplastic agents. *Int. J. Cancer*, **54**, 435–442.

TOEDIN H, STAEHELIN T AND GORDON J. (1979). Electrophoretic transfer of proteins from polyacrylamide gels to nitrocellulose sheets: procedure and some applications. *Proc. Natl Acad. Sci USA*, **76**, 4350–4353.

TONKS NK, DILTZ CD AND FISCHER EH. (1988). Characterization of the major protein-tyrosine-phosphatases of human placenta. *J. Biol. Chem.*, **263**, 6731–6737.

XUAN YZ, BHUSHAN A, HACKER MP AND TRITTON TR. (1994). Tyrosine phosphorylation of a membrane protein (P66) may play an important role in methotrexate transport, (II). *Proc. Am. Assoc. Cancer Res.*, **35**, 2523.